# One-Pot Syntheses and Characterization of Group VI Carbonyl NHC Coordination Compounds

**DOI:** 10.3390/molecules30112433

**Published:** 2025-06-02

**Authors:** Zala Stopar, Evelin Gruden, Melita Tramšek, Gašper Tavčar

**Affiliations:** Department of Inorganic Chemistry and Technology, Jožef Stefan Institute, Jamova 39, 1000 Ljubljana, Slovenia

**Keywords:** NHC carbene, coordination compound, metal carbonyl

## Abstract

The reactions of *N*-heterocyclic carbenes (IMesNHC and IPrNHC) with transition metal carbonyls of group VI (Cr(CO)_6_, Mo(CO)_6_, and W(CO)_6_) were carried out in acetonitrile in simple one-pot syntheses and led to the formation of the coordination compounds IMesNHC–Cr(CO)_5_ (**1a**), IMesNHC–Mo(CO)_5_ (**2a**), IMesNHC–W(CO)_5_ (**3a**), IPrNHC–Cr(CO)_5_ (**1b**), IPrNHC–Mo(CO)_5_ (**2b**), and IPrNHC–W(CO)_5_ (**3b**). With the exception of **1b**, the coordination compounds were formed selectively and in high yields. The method represents an effective and easy-to-perform alternative to the previously described methods for NHC–M(CO)_5_ (M = Cr, Mo, W). All prepared compounds were characterized by NMR and Raman spectroscopy. Compounds **1a**, **2a**, **3a**, and **2b** were also crystallized and structurally characterized by X-ray structure analysis. Finally, the structural features of all compounds were compared with DFT calculations of structurally optimized coordination compounds.

## 1. Introduction

In recent decades, the chemistry of *N*-heterocyclic carbenes (NHCs) has made significant progress. The ease of synthesis and the great diversity of NHCs have made them practically indispensable in numerous fields of research. As ligands, they have a strong affinity for metals, including transition metals, and form strong and stable bonds. The incorporation of NHCs allows control over the reactivity, selectivity, and catalytic efficiency of various reactions. Therefore, they are an essential part of modern organometallic chemistry. In addition, NHCs offer promising potential for the development of new bioactive organometallic coordination compounds due to their cytotoxic, antimicrobial, and anticancer properties [1,2,3].

Group VI metals (Cr, Mo, and W) have played an important, central role in the development of carbene chemistry. Procedures for the preparation of NHC coordination compounds of group VI metals have often been used as model reactions in the development of many synthetic strategies. One of the first metal–NHC coordination compounds synthesized was a Cr(0)-carbonyl compound prepared by Öfele (1968) [4]. Nowadays, the NHC-stabilized metal carbonyl compounds of group VI (NHC–M(CO)_5_; M = Cr, Mo, and W) are quite well-studied and represent a dynamic field of NHC chemistry. Some of the group VI carbonyl NHC compounds have also been tested for their catalytic activity and have been shown to be useful in a variety of reactions. For example, they have been shown to be useful for the catalytic oxidation of styrene, epoxidation of olefins, and hydrosilylation of imines and ketones, while some are even suitable for photocatalytic H_2_ production [5].

**Scheme 1:**
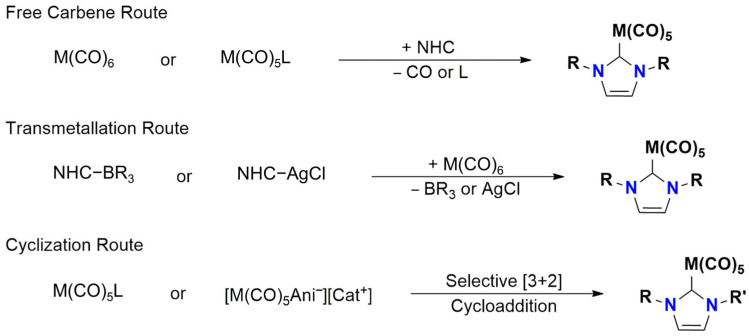
Known synthetic routs to NHCM(CO)_5_ (M = Cr, Mo, W). L = neutral ligand; Ani^−^ = anionic ligand; Cat^+^ = Cation.

In the literature, three common synthetic routes for the preparation of NHC–M(CO)_5_ compounds are described (Scheme 1). The free carbene route provides a direct, straightforward pathway for most NHC–M(0)-carbonyl coordination compounds [6,7,8]. The main advantage of this route is the use of an NHC ligand prepared in advance. An alternative method is the transmetallation route. Yamaguchi et al. [9] reported the use of NHC–BEt_3_ synthons, which can serve as precursors for the synthesis of transition metal coordination compounds. The use of a stable precursor is the main advantage of this route. Another strategy was described by Fehlharmmer et al. in 1987 [10]. The group developed a regio- and site-selective [3+2] cycloaddition reaction between the functionalized isocyano coordination compound M(CO)_5_(CNCH_2_COOEt) (M = Cr, W) and heteroallenes PhN=C=O in the presence of bases [11,12,13].

**Scheme 2:**
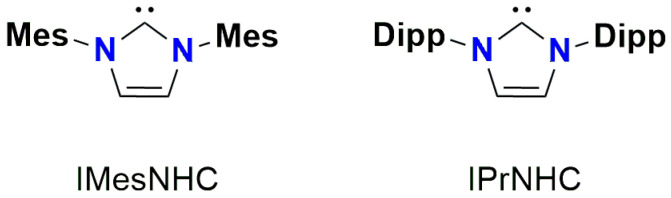
Schematic illustration of used N-heterocyclic carbenes.

In this work, we have tested the reactivity of two N-heterocyclic carbenes (IMesNHC and IPrNHC, shown in Scheme 2) with different group VI metal carbonyls (Cr(CO)_6_, Mo(CO)_6_ and W(CO)_6_). Although the chemistry of NHC–M(CO)_5_ (M = Cr, Mo, and W) compounds is quite well-known, a simple, effective, and easy-to-perform procedure for their preparation is still lacking. As far as we know, the reported yields of the above-mentioned methods are usually quite low. In this work, we have focused on an alternative process that can produce the same compounds in high yields. In addition, we have focused on the characterization of the compounds using Raman spectroscopy, which is an alternative to the commonly used IR spectroscopy.

Another aspect of our research focused on the crystallization and the structural characterization of compounds that have not yet been structurally characterized. As far as we know, only a few crystal structures of the NHC-stabilized group VI metal carbonyls have been described in the literature [14,15,16]. More specifically, of the compounds we are interested in, only two examples with IPrNHC carbenes have been described so far (IPrNHC–Cr(CO)_5_ [7] and IPrNHC–W(CO)_5_ [17]).

**Note:** This work is partially based on the master thesis defended by Z. Stopar (2024) at the Faculty of Chemistry and Chemical Technology, University of Ljubljana.

## 2. Results and Discussion

### 2.1. Synthesis and Characterization

NHC carbenes (IMesNHC and IPrNHC) reacted with carbonyls of group VI metals (Cr(CO)_6_, Mo(CO)_6_, and W(CO)_6_) in MeCN to form the corresponding coordination compounds NHC–M(CO)_5_ (M = Cr, Mo, W). The synthetic procedures and the characterization of all coordination compounds are presented in the following chapters, while the crystallographic data for the structurally characterized compounds are given in the Supporting Information.

#### Coordination Compounds with Metal Hexacarbonyls (M(CO)_6_; M = Cr, Mo, W)

**Scheme 3:**
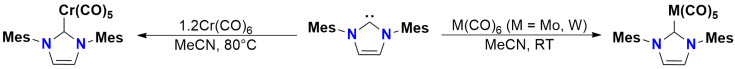
Synthesis of IMesNHC–M(CO)_5_ (M = Cr, Mo, W). Mes = 2,3,5-trimethylphenyl.

First, the coordination compounds were prepared with IMesNHC. Cr(CO)_6_, Mo(CO)_6_, and W(CO)_6_ reacted with free IMesNHC to form IMesNHC–Cr(CO)_5_ (**1a**), IMesNHC–Mo(CO)_5_ (**2a**), and IMesNHC–W(CO)_5_ (**3a**), respectively. All reactions were carried out in MeCN for 16 h and quantitatively formed pale yellow solids. While Mo(CO)_6_ and W(CO)_6_ already reacted at room temperature, the reaction with Cr(CO)_6_ was heated to 80 °C to achieve a complete conversion (Scheme 3).

The formation of the desired products was confirmed by NMR spectrometry (^1^H and ^13^C NMR spectra of compounds **1a**, **2a,** and **3a** are available in the Supporting Information). All spectra obtained are in good agreement with the literature data of the known compounds. A comparison of the ^13^C NMR peaks of the prepared compounds with similar known compounds is given in Table S1 in the Supporting Information. The reactions were simple and easy to carry out, and required no additional purification. All products were obtained in high yields. In addition, the compounds were successfully crystallized and structurally characterized.

In the past, IMesNHC–Cr(CO)_5_, IMesNHC–Mo(CO)_5_, and IMesNHC–W(CO)_5_ have already been prepared using different procedures. IMesNHC–Cr(CO)_5_ was synthesized by Chung’s group in 2007 by the reaction of chromium Fischer carbene ([Cr{=OMe(R)}(CO)_5_]) with an NHC ligand, with the Fischer carbene serving as the source of chromium carbonyl [7]. In 2004, the IMesNHC–Mo(CO)_5_ was prepared by using IMesNHC–BEt_3_ synthon as a IMesNHC precursor in the transmetallation reaction with Mo(CO)_6_ [9], while the preparation of IMesNHC–W(CO)_5_ involved silver-mediated transmetallation with silver carbene formed in situ [8]. After purification, the compounds were obtained in moderate yields (Cr: 67% [7], Mo: 67% [9], and W: 60% [8]). Our procedure represents a simpler, modified synthetic route that can be carried out in one pot and leads to products with high yields that do not require additional purification (yields **1a**: 91%, **2a**: 85%, and **3a**: 96%).

**Scheme 4:**
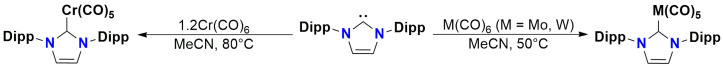
Synthesis of IPrNHC–M(CO)_5_ (M = Cr, Mo, W). Dipp = 2,5-diisopropylphenyl.

Next, the coordination compounds were prepared with IPrNHC. The M(CO)_6_ (M = Cr, Mo, W) reacted with free IPrNHC to afford IPrNHC–Cr(CO)_5_ (**1b**), IPrNHC–Mo(CO)_5_ (**2b**), and IPrNHC–W(CO)_5_ (**3b**). All reactions were successfully carried out in MeCN for 16 h as shown in Scheme 4. Compared to the IMesNHC counterparts, the reactions for the IPrNHC required higher temperatures. The reactions with Mo(CO)_6_ and W(CO)_6_ were carried out at 50 °C for 16 h to achieve quantitative yields. However, the reaction with Cr(CO)_6_ did not progress as efficiently as the reactions with the carbonyls of its cogeners. The reaction with 1.2 eq. Cr(CO)_6_ was heated to 80 °C and still did not result in complete conversion. The mixture still contained some unreacted IPrNHC, which was washed out with hexane. Despite trying, we were not able to obtain better reaction yields for **1b** even when increasing the amount of Cr(CO)_6_ up to 1.5 eq.

After the removal of the solvents under a vacuum, the three compounds were formed as pale yellow solids in moderate to high yields (**1b**: 48%, **2b**: 95%, and **3b**: 92%). The formation of the desired products was confirmed by NMR spectrometry (^1^H and ^13^C NMR spectra of compounds **1b**, **2b**, and **3b** can be found in the Supporting Information). All spectra obtained are in good agreement with the literature data of the known compounds. A comparison of the ^13^C NMR peaks of the prepared compounds with similar known compounds is given in Table S1 in the Supporting Information.

In the past, IPrNHC–Cr(CO)_5_, IPrNHC–Mo(CO)_5_, and IPrNHC–W(CO)_5_ have already been prepared using different methods. All three were prepared in 2007 by Chung’s group, by the reaction of chromium Fischer carbenes ([M{=OMe(R)}(CO)_5_]; M = Cr, Mo, W) with an NHC ligand [7]. The reactions proceeded in moderate to high yields (Cr: 62%, Mo: 91%, and W: 40%). The group also succeeded in structurally characterizing IPrNHC–Cr(CO)_5_ [7]. A few years later, IPrNHC–Cr(CO)_5_ and IPrNHC–W(CO)_5_ were prepared in a two-step reaction by the group of Rivard [18,19]. First, the THF–W(CO)_6_ adduct was formed by irradiating W(CO)_6_ in THF with a mercury lamp. Later, the IPrNHC was added to form the IPrNHC–W(CO)_5_ complex [18]. The same method was used by the group of Ghadwal and Frenking to prepare and structurally characterize the IPrNHC–W(CO)_5_ [17]. Our method represents an alternative route to the group VI NHC stabilized carbonyl compounds that does not require photochemical reaction conditions but only light heat treatment.

### 2.2. Crystal Structure Analysis

**Figure 1:**
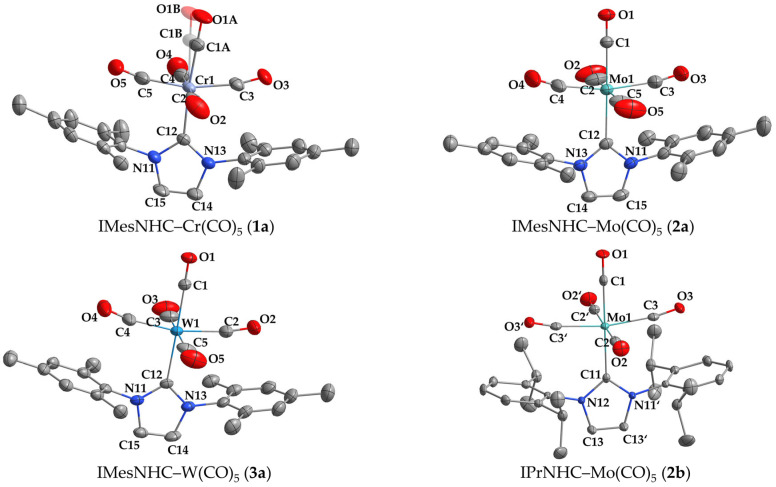
Crystal structures of IMesNHC–Cr(CO)_5_ (**1a**), IMesNHC–Mo(CO)_5_ (**2a**), IMesNHC–W(CO)_5_ (**3a**), and IPrNHC–Mo(CO)_5_ (**2b**) (Symmetry code: (i) 1 − x, y, 3/2 – z). The ellipsoids are drawn at 50% probability. The positions of the disordered atoms are shown in domain A and B. For clarity, all hydrogen atoms are omitted and the domain B is shaded.

Single crystals of IMesNHC–Cr(CO)_5_ (**1a**) were obtained by slow solvent evaporation from saturated Et_2_O solution, while IMesNHC–Mo(CO)_5_ (**2a**) and IMesNHC–W(CO)_5_ (**3a**) crystallized by slow solvent evaporation from saturated MeCN solutions and formed single crystals of IMesNHC–Mo(CO)_5_·MeCN (**2a**·MeCN) and IMesNHC–W(CO)_5_·MeCN (**3a**·MeCN), respectively. Similarly, the single crystals of IPrNHC–Mo(CO)_5_ (**2b**) were prepared by slow solvent evaporation from a saturated MeCN solution. The crystal structures of **1a**, **2a**, **3a**, and **2b** are shown in Figure 1.

IMesNHC–Cr(CO)_5_ (**1a**) crystallizes in the monoclinic space group P2_1_/n, while structurally related compounds IMesNHC–Mo(CO)_5_ (**2a**) and IMesNHC–W(CO)_5_ (**3a**) co-crystallize with a MeCN molecule in the monoclinic space group P2_1_/c. The related IPrNHC–Mo(CO)_5_ (**2b**) crystallizes in the orthorhombic space group Cmcm. In all cases, the central metal atoms are hexa-coordinated and have five CO ligands and one NHC ligand. In all crystal structures, the NHC ligand is eclipsed to the equatorial metal tetracarbonyl fragment. The eclipsed carbonyl groups are strongly bent (average M–C–O angle in **2a**: 170.0°) compared to other carbonyl groups (average M–C–O angle in **2a**: 177.8°). This trend is consistent with the literature [7]. One reason for this is probably the presence of intramolecular contacts between the eclipsed carbonyl groups and the aromatic rings. According to the literature, these non-covalent, weakly attractive interactions can be classified as n → π* interactions or *π* → π* contacts [20,21]. In addition, we observed that the C≡O bond lengths of the eclipsed carbonyl groups are slightly longer than the non-eclipsed bonds (eclipsed C3–O3 in **2b**: 1.1406(3) Å; non-eclipsed C2–O2 in **2b**: 1.1394(3) Å), which may be due to the interactions between the two fragments [21].

In IMesNHC–Cr(CO)_5_ (**1a**), the asymmetric unit contains two octahedrally coordinated coordination compounds, while the asymmetric units of IMesNHC–Mo(CO)_5_·MeCN (**2a**·MeCN) and IMesNHC–W(CO)_5_·MeCN (**3a**·MeCN) both contain a disordered MeCN molecule. The asymmetric units of **1a**, **2a**·MeCN, and **3a**·MeCN are shown in the Supporting Information. On the other hand, the asymmetric unit of IPrNHC–Mo(CO)_5_ (**2b**) contains only half of the coordination compound because the crystal structure has a mirror plane passing through C(11), Mo(1), C(1), and O(1) atoms.

The average M–C_(carbene)_ bond length of IMesNHC–Cr(CO)_5_ (**1a**) (2.1371 Å) is consistent with the bond length in its IPrNHC and other NHC analogs (2.142(2) Å [7], 2.125(3) Å [15], and 2.131(2) Å [16]). The average M–CO_(trans)_ bond length of compound **1a** (1.849 Å) is also in good agreement with the literature data of structurally related compounds (1.843(5) Å [7], 1.857(3) Å [15], and 1.868(2) Å [16]). The CO–Mo–C_(carbene)_ angle of compound **1a** (175.4°) is in good agreement with structurally related NHC compounds with slightly bent CO–Mo–C_(carbene)_ (179.9(2)° [7], 176.16(14)° [15]), while the analogous angle of a structurally related coordination compound is straight due to its position on the mirror plane (180.0° [16]). Similar to **1a**, the CO–Mo–C_(carbene)_ angles of IPrNHC and other structurally related NHC compounds are also slightly bent (177.3(2)° [15] and 176.3(1)° [22]).

The M–C_(carbene)_ bond length of IMesNHC–Mo(CO)_5_ (**2a**) (2.278(2) Å) is comparable to the bond length of IPrNHC–Mo(CO)_5_ (**2b**) (2.264(3) Å). Moreover, both bond lengths are in agreement with the literature data for coordination compounds with a Mo(CO)_5_ moiety (2.288(9) Å [14] and 2.257(6) Å [15]). The Mo–CO_(trans)_ bond length between the Mo atom and the CO ligand in a trans position relative to the NHC is 1.987(2) Å for compound **2a** and 1.985(3) Å for **2b**. These values are also in good agreement with the Mo–CO_(trans)_ bond length of analogous NHC–Mo(CO)_5_ derivatives (2.00(1) Å and 1.993(65) Å [15]). Moreover, the CO–Mo–C_(carbene)_ bond angles of **2a** (179.1(1)°) and **2b** (180.0°) are similar as in the literature for NHC–Mo(CO)_5_ derivatives (178.1(1)° [14] and 175.686(231)° [15]).

The M–C_(carbene)_ bond length of IMesNHC–W(CO)_5_ (**3a**) (2.264(3) Å) agrees with the bond length in its IPrNHC and other NHC analogs (2.260(2) Å [17], 2.259(4) Å [17], and 2.272(3) Å [22]). The M–CO_(trans)_ bond length of compound **3a** (1.990(4) Å) is also in good agreement with the literature data of structurally related compounds (1.997(2) Å [17], 1.994(6) Å [17], and 1.999(4) Å [22]). The CO–Mo–C_(carbene)_ angle of compound **3a** is (179.0(1)°), while the analogous angle in the coordination compound IPrNHC–W(CO)_5_ is straight (180.0° [17]), which is due to its position on the mirror plane. Similar to **3a**, the CO–Mo–C_(carbene)_ angles of the structurally related NHC compounds are also slightly bent (177.3(2)° [15] and 176.3(1)° [22]).

### 2.3. Raman Spectroscopy Results

**Figure 2:**
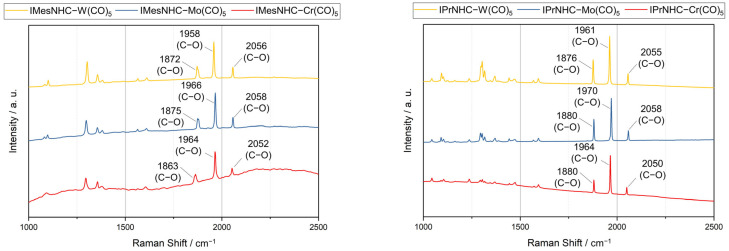
Raman spectra of IMesNHC–Cr(CO)_5_ (**1a**), IMesNHC–Mo(CO)_5_ (**2a**), and IMesNHC–W(CO)_5_ (**3a**) (**left**), and IPrNHC–Cr(CO)_5_ (**1b**), IPrNHC–Mo(CO)_5_ (**2b**), and IPrNHC–W(CO)_5_ (**3b**) (**right**) with assigned C–O vibrations.

Raman spectroscopy was used for the characterization of the compounds as an alternative method to IR spectroscopy. The complete Raman spectra of the prepared compounds can be found in the Supporting Information. Figure 2 shows the spectra of NHC–M(CO)_5_ (NHC = IMesNHC, IPrNHC; M = Cr, Mo, W) with the assigned C–O vibrations.

Three strong carbonyl bands in the range of 1500–2100 cm^−1^ were observed for all compounds. A comparison of the experimentally determined Raman shifts with the IR bands of the carbonyl groups described in the literature for each coordination compound is shown in Table 1. As can be seen from the table, the experimentally determined Raman shifts are in good agreement with the IR spectroscopic data reported in the literature. A similar observation can also be found in a 1990 study in which the authors reported the Raman shifts and IR bands of the C–O vibrations for the (*η*^6^-mesitylene)M(CO)_3_ carbonyl compounds of group VI. The reported values of the Raman shifts were found to be similar to the values of the IR bands [23]. Therefore, we conclude that the IR bands of NHC-stabilized group VI carbonyl coordination compounds are comparable to the Raman shifts.

### 2.4. Computational Results

**Figure 3:**
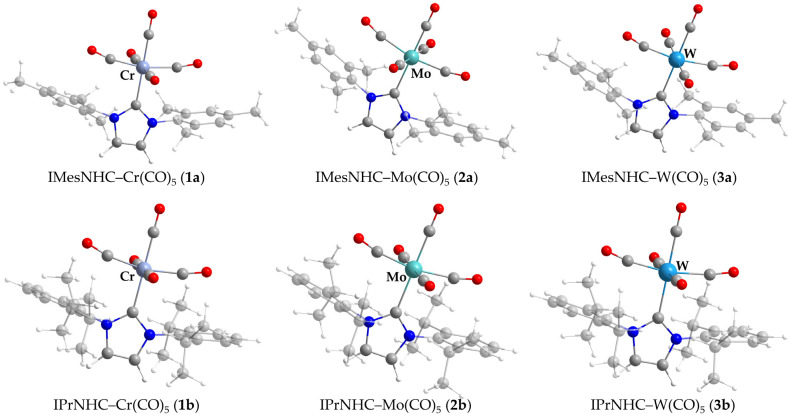
Optimized structures of IMesNHC and IPrNHC coordination compounds.

The structures of (IMesNHC–Cr(CO)_5_ (**1a**), IMesNHC–Mo(CO)_5_ (**2a**), IMesNHC–W(CO)_5_ (**3a**), IPrNHC–Cr(CO)_5_ (**1b**), IPrNHC–Mo(CO)_5_ (**2b**), and IPrNHC–W(CO)_5_ (**3b**) were used as starting points for the optimization of the DFT calculations. Geometry optimizations and frequency calculations were performed. The optimized structures were further compared with the experimentally obtained coordination compounds. Figure 3 shows the optimized structures of the coordination compounds. A comparison between the experimental and calculated bond distances of the coordination compounds is shown in Table 2. The calculated M–C_(carbene)_ bond lengths of the metal–carbonyl coordination compounds (2.271 Å; **3a**) are in good agreement with the experimental results (2.264(3) Å; **3a**). The differences between the calculated and experimental values can be attributed to the fact that the calculations were performed in the gas phase, where the effect of crystal packing was neglected. The bending of eclipsed carbonyls is also observed and it is in agreement with experimental data.

The estimated enthalpies and Gibbs free energies for the formation of metal–carbonyl coordination compounds were calculated according to the following chemical reaction:

$$\mathrm{NHC}+M{\left(\mathrm{CO}\right)}_{6}\;\to \;\mathrm{NHC}–M{\left(\mathrm{CO}\right)}_{5}+\mathrm{CO}$$

The calculated enthalpies and Gibbs free energies of reactions of the optimized compounds can be found in the Supporting Information. For both values, the trend within the series is similar. In all series, for both IMesNHC and IPrNHC ligands, the calculated enthalpies of reactions are exothermic. The calculated enthalpies of reactions for each compound in the NHC–M(CO)_5_ (M = Cr, Mo, W) series become more exothermic as the metal becomes heavier. For example, the estimated enthalpy for the formation of IMesNHC–Cr(CO)_5_ is −62.18 kJ/mol, while, for IMesNHC–W(CO)_5_, it is −74.32 kJ/mol. It seems that the reactions with heavier metals of group VI are favored compared to their lighter relatives. This agrees well with our experimental results, as a higher reaction temperature (80 °C) was required for the formation of Cr coordination compounds than for the Mo and W counterparts (up to 50 °C).

## 3. Materials and Methods

### 3.1. Reagents

Commercially available reagents Cr(CO)_6_ (Alfa Aesar GmbH & Co KG, Haverhill, MA, ZDA, 100%), Mo(CO)_6_ (Sigma-Aldrich, St. Louis, MO, USA, 98%), and W(CO)_6_ (Sigma-Aldrich, 97%) were used as received. The IPrNHC carbene was synthesized by deprotonation of 1,3-bis(1,3-diisopropy)imidazolium chloride according to the procedures described in the literature [24], while the IMesNHC carbene was synthesized by deprotonation of 1,3-bis(2,4,6-trimethylphenyl)imidazolium chloride with potassium bis(trimethylsilyl)amide (Thermo Fisher Scientific, Waltham, MA, USA, 0.7M solution in toluene) according to the modified procedures described below (Section 3.3.1). Both chloride salts were prepared according to the literature procedure [25]. Solvents MeCN (Honeywell, Charlotte, NC, USA, ≥99.9%), Et_2_O (Sigma-Aldrich, ≥99.9%), and hexane (Honeywell, ≥97.0%) were purified using the Vigor solvent purification system (Vigor Tech, Houston, TX, USA). Deuterated solvent C_6_D_6_ (Deutero, Kastellaun, Germany, 99.0%) were stored in glovebox (M. Braun, Garching bei Munchen, Germany) over activated 3 Å molecular sieves before use. Molecular sieves were activated by heating at 150 °C in vacuo overnight.

### 3.2. General

All syntheses were performed under an argon atmosphere using Schlenk and glovebox techniques. Commercially available reagents and synthesized carbene ligands were stored in a glovebox (M. Braun) maintained below 0.1 ppm O_2_ and H_2_O. All reactions were performed in 100 mL Schlenk flasks. Crystallization of prepared coordination compounds proceeded by evaporation of solvents from a saturated solution.

### 3.3. Syntheses

#### 3.3.1. Modified Synthesis of IMesNHC Carbene

To a stirred solution of 1,3-bis(2,4,6-trimethylphenyl)imidazolium chloride (5 g, 15 mmol) in THF (250 mL) was added potassium bis(trimethylsilyl)amide (0.7M, 21 mL, 0.15 mmol) at −78 °C. The mixture was allowed to warm to room temperature over a period of 3 h. Then, the solution was filtered through Celite to remove KCl and all volatiles were removed under vacuum (3.8 g, 85% yield). The formation of the IMesNHC carbene was confirmed by NMR spectroscopy. ^1^H NMR (C_6_D_6_, 25 °C, 600.06 MHz): *δ* 6.81 (s, 4H, *m*-Ar***H***), 6.49 (s, 2H, C***H***=C***H***), 2.16 (s, 18H, *o*-CH_3_ & *p*-CH_3_).

#### 3.3.2. IMesNHC–Cr(CO)_5_ (**1a**)

A suspension of IMesNHC (0.150 g, 0.5 mmol) and Cr(CO)_6_ (0.130 g, 0.6 mmol) was stirred in MeCN (12 mL) for 16 h at 80 °C. Solvent was then removed under vacuum. The isolated product was pale-yellow powder (0.221 g, 91% yield). Single crystals were prepared by slow solvent evaporation from a saturated solution of IMesNHC–Cr(CO)_5_ in Et_2_O. ^1^H NMR (C_6_D_6_, 25 °C, 600.06 MHz): *δ* 6.76 (s, 4H, *m*-Ar***H***), 6.11 (s, 2H, C***H***=C***H***), 2.13 (s, 6H, *p*-C***H***_3_), 2.01 (s, 12H, *o*-C***H***_3_). ^13^C{^1^H} NMR (C_6_D_6_, 25 ^°^C, 150.89 MHz): 221.5 (*trans-**C***O), 217.3 (*cis-**C***O), 197.7 (***C***-Cr(CO)_5_), 139.9 (Ar***C***), 137.9 (Ar***C***), 135.8 (Ar***C***), 129.8 (Ar***C***), 124.1 (***C***H=***C***H), 21.1 (***C***H_3_), 17.7 (***C***H_3_). Crystal Data for C_26_H_24_CrN_2_O_5_ (M = 496.47 g/mol): monoclinic, space group P2_1_/n (no. 14), a = 17.1956(3) Å, b = 17.3891(2) Å, c = 17.7965(3) Å, β = 109.157(2)°, V = 5026.8(2) Å^3^, Z = 8, T = 150 K, μ(CuKα) = 4.206 mm^−1^, Dcalc = 1.312 g/cm^3^, 34,016 reflections measured (3.5 ≤ 2Θ ≤ 76.3), and 10,413 unique (Rint = 0.0316, Rsigma = 0.0325) which were used in all calculations. The final R1 was 0.0407 (I > 2σ(I)) and wR2 was 0.1204 (all data). Raman [ν(Cr–CO) range]: ν = 1863 cm^−1^, 1964 cm^−1^, 2052 cm^−1^.

#### 3.3.3. IMesNHC–Mo(CO)_5_ (**2a**)

A suspension of IMesNHC (0.150 g, 0.5 mmol) and Mo(CO)_6_ (0.130 g, 0.5 mmol) was stirred in MeCN (12 mL) for 16 h at room temperature. Solvent was then removed under vacuum. The isolated product was pale-yellow powder (0.226 g, 85% yield). Single crystals were prepared by slow solvent evaporation from a saturated solution of IMesNHC–Mo(CO)_5_ in MeCN. ^1^H NMR (C_6_D_6_, 25 °C, 600.06 MHz): *δ* 6.77 (s, 4H, *m*-Ar***H***), 6.11 (s, 2H, C***H***=C***H***), 2.13 (s, 6H, *p*-C***H***_3_), 1.98 (s, 12H, *o*-C***H***_3_). ^13^C{^1^H} NMR (C_6_D_6_, 25 °C, 150.89 MHz): 211.5 (*trans-**C***O), 206.1 (*cis-**C***O), 193.9 (***C***-Mo(CO)_5_), 139.8 (Ar***C***), 138.2 (Ar***C***), 135.6 (Ar***C***), 129.8 (Ar***C***), 123.2 (***C***H=***C***H), 21.1 (***C***H_3_), 17.7 (***C***H_3_). Raman [ν(Mo–CO) range]: ν = 1875 cm^−1^, 1965 cm^−1^, 2058 cm^−1^. Crystal Data for C_28_H_27_MoN_3_O_5_ (M = 581.46 g/mol): monoclinic, space group P2_1_/c (no. 14), a = 8.6053(2) Å, b = 15.6382(3) Å, c = 21.0346(3) Å, β = 101.407(2)°, V = 2774.74(9) Å^3^, Z = 4, T = 150 K, μ(CuKα) = 4.206 mm^−1^, Dcalc = 1.392 g/cm^3^, 44,450 reflections measured (3.5 ≤ 2Θ ≤ 76.3), and 5798 unique (Rint = 0.0490, Rsigma = 0.0272) which were used in all calculations. The final R1 was 0.0342 (I > 2σ(I)) and wR2 was 0.0877 (all data).

#### 3.3.4. IMesNHC–W(CO)_5_ (**3a**)

A suspension of IMesNHC (0.150 g, 0.5 mmol) and W(CO)_6_ (0.173 g, 0.5 mmol) was stirred in MeCN (12 mL) for 16 h at room temperature. Solvent was then removed under vacuum. The isolated product was pale-yellow powder (0.295 g, 96% yield). Single crystals were prepared by slow solvent evaporation from a saturated solution of IMesNHC–W(CO)_5_ in MeCN. ^1^H NMR (C_6_D_6_, 25 °C, 600.06 MHz): δ 6.76 (s, 4H, *m*-Ar***H***), (s, 2H, C***H***=C***H***), (s, 6H, *p*-C***H***_3_), (s, 12H, *o*-C***H***_3_). ^13^C{^1^H} NMR (C_6_D_6_, 25 ^°^C, 150.89 MHz): 197.7 (***C***O), 139.9 (Ar***C***), 138.1 (Ar***C***), 135.6 (Ar***C***), 129.9 (Ar***C***), 123.1 (***C***H=***C***H), 21.1 (***C***H_3_), 17.9 (***C***H_3_). Raman [ν(W–CO) range]: ν = 1872 cm^−1^, 1958 cm^−1^, 2056 cm^−1^. Crystal Data for C_28_H_27_WN_3_O_5_ (M = 669.37 g/mol): monoclinic, space group P2_1_/c (no. 14), a = 8.6040(2) Å, b = 15.6386(2) Å, c = 20.9235(3) Å, β = 101.641(2)°, V = 2757.44(9) Å^3^, Z = 4, T = 150 K, μ(CuKα) = 8.105 mm^−1^, Dcalc = 1.612 g/cm^3^, 28,855 reflections measured (2.8 ≤ 2Θ ≤ 76.3), and 5757 unique (Rint = 0.0507, Rsigma = 0.0378) which were used in all calculations. The final R1 was 0.0310 (I > 2σ(I)) and wR2 was 0.0795 (all data).

#### 3.3.5. IPrNHC–Cr(CO)_5_ (**1b**)

A suspension of IPrNHC (0.200 g, 0.5 mmol) and Cr(CO)_6_ (0.130 g, 0.6 mmol) was stirred in MeCN (12 mL) for 16 h at 80 °C. Solvent was then removed under vacuum, and the product was washed 3 times with 1 mL hexane. The isolated product was pale-yellow powder (0.134 g, 48% yield). ^1^H NMR (C_6_D_6_, 25 °C, 600.06 MHz): *δ* 7.26 (t, 2H, *J* = 7.7 Hz, *p*-Ar***H***), 7.13 (d, 4H, *J* = 7.7 Hz, *m*-Ar***H***), 6.52 (s, 2H, C***H***=C***H***), 2.79 (sept, 4H, *J* = 6.8 Hz, C***H***(CH_3_)_2_), 1.40 (d, 12H, *J* = 6.8 Hz, CH(C***H_3_***)_2_), 0.99 (d, 12H, *J* = 6.8 Hz, CH(C***H_3_***)_2_). ^13^C{^1^H} NMR (C_6_D_6_, 25 °C, 150.89 MHz): 221.0 (*trans-**C***O), 216.8 (*cis-**C***O), 200.4 (***C***-Cr(CO)_5_), 146.4 (*ipso*-Ar), 137.9 (Ar***C***), 131.2 (Ar***C***), 125.6 (***C***H=***C***H), 124.7 (***C***H=***C***H), 28.9 (***C***H(CH_3_)_2_), 26.0 (CH(***C***H_3_)_2_), 22.8 (CH(***C***H_3_)_2_). Raman [ν(Cr–CO) range]: ν = 1880 cm^−1^, 1964 cm^−1^, 2050 cm^−1^.

#### 3.3.6. IPrNHC–Mo(CO)_5_ (**2b**)

A suspension of IPrNHC (0.200 g, 0.5 mmol) and Mo(CO)_6_ (0.136 g, 0.5 mmol) was stirred in MeCN (12 mL) for 16 h at 50 °C. Solvent was then removed under vacuum. The isolated product was pale-yellow powder (0.305 g, 95% yield). Single crystals were prepared by slow solvent evaporation from a saturated solution of IPrNHC–Mo(CO)_5_ in MeCN. ^1^H NMR (C_6_D_6_, 25 °C, 600.06 MHz): δ 7.27 (t, 2H, *J* = 7.8 Hz, *p*-Ar***H***), 7.13 (d, 4H, *J* = 7.8 Hz, *m*-Ar***H***), 6.49 (s, 2H, C***H***=C***H***), 2.78 (sept, 4H, *J* = 6.9 Hz, C***H***(CH_3_)_2_), 1.38 (d, 12H, *J* = 6.8 Hz, CH(C***H***_3_)_2_), 0.99 (d, 12H, *J* = 6.9 Hz, CH(C***H***_3_)_2_). ^13^C{^1^H} NMR (C_6_D_6_, 25 °C, 150.89 MHz): 211.0 (*trans-**C***O), 205.7 (*cis-**C***O), 196.9 (***C***-Mo(CO)_5_), 146.2 (*ipso*-Ar), 138.1 (Ar***C***), 131.1 (Ar***C***), 124.8 (***C***H=***C***H), 124.7 (***C***H=***C***H), 28.9 (***C***H(CH_3_)_2_), 25.9 (CH(***C***H_3_)_2_), 22.8 (CH(***C***H_3_)_2_). Raman [ν(Mo–CO) range]: ν = 1880 cm^−1^, 1970 cm^−1^, 2058 cm^−1^. Crystal Data for C_32_H_36_MoN_2_O_5_ (M = 624.57 g/mol): orthorombic, space group Cmcm (no. 63), a = 11.2283(5) Å, b = 13.9658(7) Å, c = 19.7145(9) Å, V = 3091.5(3) Å^3^, Z = 4, T = 150 K, μ(MoKα) = 0.465 mm^−1^, Dcalc = 1.342 g/cm^3^, 7484 reflections measured (3.6 ≤ 2Θ ≤ 28.8), and 1875 unique (Rint = 0.0284, Rsigma = 0.0255) which were used in all calculations. The final R1 was 0.0228 (I > 2σ(I)) and wR2 was 0.0555 (all data).

#### 3.3.7. IPrNHC–W(CO)_5_ (**3b**)

A suspension of IPrNHC (0.250 g, 0.6 mmol) and Mo(CO)_6_ (0.227 g, 0.6 mmol) was stirred in MeCN (12 mL) for 16 h at 50 °C. Solvent was then removed under vacuum. The isolated product was pale-yellow powder (0.337 g, 92% yield). ^1^H NMR (C_6_D_6_, 25 °C, 600.06 MHz): δ 7.27 (t, 2H, *J* = 7.8 Hz, *p*-Ar***H***), 7.12 (d, 4H, *J* = 7.8 Hz, *m*-Ar***H***), 6.47 (s, 2H, C***H***=C***H***), 2.76 (sept, 4H, *J* = 6.8 Hz, *C**H***CH_3_), 1.38 (d, 12H, *J* = 6.8 Hz, CH(C***H***_3_)_2_), 0.99 (d, 12H, *J* = 6.8 Hz, CH(C***H***_3_)_2_). ^13^C{^1^H} NMR (C_6_D_6_, 25 °C, 150.89 MHz): 199.9 (*trans-**C***O), 197.3 (*cis-**C***O), 188.7 (***C***-W(CO)_5_), 146.2 (*ipso*-Ar), 138.0 (Ar***C***), 131.2 (Ar***C***), 124.7 (***C***H=***C***H), 29.1 (***C***H(CH_3_)_2_), 25.8 (CH(***C***H_3_)_2_), 22.8 (CH(***C***H_3_)_2_). Raman [ν(W–CO) range]: ν = 1880 cm^−1^, 1970 cm^−1^, 2055 cm^−1^.

### 3.4. NMR Spectroscopy

Samples were prepared under inert atmosphere in a glovebox (M. Braun). NMR spectra were recorded in 5 mm glass NMR tubes. Measurements were performed at the Slovenian NMR Centre (National Institute of Chemistry, Ljubljana, Slovenia) using a Bruker AVANCE NEO 600 MHz NMR spectrometer (Bruker Coorporation, Billerica, MA, USA). Chemical shifts of ^1^H and ^13^C were referenced to residual signals of deuterated solvents (CDCl_3_, MeCN-d_3_, C_6_D_6_) and reported relative to TMS (tetramethylsilane) [26].

### 3.5. Crystal Structure Determination

Crystal data for all compounds were collected with a Gemini A diffractometer (Agilent Technologies, Santa Clara, CA, USA) equipped with an Atlas CCD detector using graphite-monochromated Cu or Mo Kα radiation at 150 K. The data were processed using the CrysAlisPro software package [27]. An analytical absorption correction was applied to all data sets [28]. Structures were solved using the SHELXT program [29]. Structure refinement was performed using the SHELXT software [30] implemented in the Olex2 program package [31]. The figures were created using Diamond 4.0 [32].

CCDC 2448746-2448749 contains the supplementary crystallographic data for this paper. These data can be obtained free of charge via https://www.ccdc.cam.ac.uk/, accessed on 1 June 2025 (or from the CCDC, 12 Union Road, Cambridge CB2 1EZ, UK; Fax: +44 1223 336033; E-mail: deposit@ccdc.cam.ac.uk).

### 3.6. Raman Spectroscopy

Samples were filled into 0.3 mm quartz capillaries under an inert atmosphere in a glovebox (M. Braun). Raman spectra were recorded using a Horiba Jobin Yvon Labram-HR spectrometer (HORIBA, Ltd., 2 Miyanohigashi, Kisshion, Minami-ku Kyoto, 601-8510 Japan) coupled with an Olympus BXFM-ILHS microscope (Olympus Corporation, Shinjuku, Tokyo, Japan) at room temperature. Samples were excited with the 633 nm emission line of a He–Ne laser and 532 nm emission line of a Nd:YAG laser.

### 3.7. Molecular Calculations

Molecular gas-phase calculations were performed with the Gaussian 16 program [33] using the Perdew–Burke–Ernzerhof (PBE) exchange–correlation functional [34] and a D3 empirical dispersion correction of Grimme [35] with Becke–Johnson damping [36]. We used the triple-ζ basis set with polarization functions, in particular, Def2-TZVP [37,38].

## 4. Conclusions

In this study, we systematically introduce a simple and straightforward one-pot synthetic procedure for preparing NHC-stabilized group VI metal carbonyl coordination compounds. By reacting commercially available group VI metal carbonyls, M(CO)_6_ (where M represents Cr, Mo, and W) with NHC ligands (IMesNHC and IPrNHC), we synthesized a series of coordination compounds. The optimal solvent for these reactions was found to be MeCN, leading to the successful formation of the coordination compounds IMesNHC–Cr(CO)_5_ (**1a**), IMesNHC–Mo(CO)_5_ (**2a**), IMesNHC–W(CO)_5_ (**3a**), IPrNHC–Cr(CO)_5_ (**1b**), IPrNHC–Mo(CO)_5_ (**2b**), and IPrNHC–W(CO)_5_ (**3b**). Except for compound **1b**, all were obtained in high yields without requiring additional purification. Notably, heating was necessary for reactions involving IPrNHC, whereas, for IMesNHC, heating was only required for the formation of **1a**. The described method represents an effective procedure with high yield compared to previously described methods for the preparation of NHC-supported group VI carbonyl compounds, which usually require more experimentally demanding techniques, such as the use of Fischer carbenes, transmetallation procedures, or UV light activation. Additionally, intramolecular contacts between the carbonyl groups and the aromatic rings were observed in the crystal structures of the synthesized compounds. For the first time, X-ray structure analysis determined the crystal structures of compounds **1a**, **2a**, **3a**, and **2b**. The structural characteristics of all compounds aligned well with the DFT calculations. Furthermore, Raman spectroscopy was successfully tested as an effective alternative to the more commonly used IR spectroscopy, even without special sample preparation.

## Figures and Tables

**Table 1:** Selected spectroscopic data for NHC coordination compounds.

	Experimental	Literature	
Compound	Raman (cm^−1^)	IR (cm^−1^)	Reference
IMesNHC–Cr(CO)_5_ (**1a**)	1863, 1964, 2052	1917, 2044	[7]
IMesNHC–Mo(CO)_5_ (**2a**)	1875, 1966, 2058	1879, 1924, 2059	[8]
IMesNHC–W(CO)_5_ (**3a**)	1872, 1958, 2056	1876, 1911, 2057	[8]
IPrNHC–Cr(CO)_5_ (**1b**)	1880, 1964, 2050	1880, 1918, 1981, 2056	[19]
IPrNHC–Mo(CO)_5_ (**2b**)	1880, 1970, 2058	1924, 2060	[7]
IPrNHC–W(CO)_5_ (**3b**)	1876, 1961, 2055	1880, 1918, 1981, 2056	[18]

**Table 2:** Selected bond lengths (Å) for NHC coordination compounds.

	M–C_(carbene)_ (Å)		*trans*-CO–M (Å)	
Compound	Experimental	Calculated	Experimental	Calculated
IMesNHC–Cr(CO)_5_ (**1a**)	2.142(2) *	2.131	1.847(2) *	1.853
IMesNHC–Mo(CO)_5_ (**2a**)	2.278(2)	2.273	1.987(2)	2.000
IMesNHC–W(CO)_5_ (**3a**)	2.264(3)	2.271	1.990(4)	2.018
IPrNHC–Cr(CO)_5_ (**1b**)	2.142(4) [7]	2.119	1.843(5) [7]	1.854
IPrNHC–Mo(CO)_5_ (**2b**)	2.264(3)	2.259	1.985(3)	2.003
IPrNHC–W(CO)_5_ (**3b**)	2.260(2) [17]	2.259	1.997(2) [17]	2.020

* The asymmetric unit of IMesNHC–Cr(CO)_5_ (**1a**) contains two molecules of the coordination compounds. For this purpose, the bond lengths of Molecule 2 are shown.

## Data Availability

The data presented in this study are available upon request from the corresponding author.

## Supplementary Materials

The following supporting information can be downloaded at: https://www.mdpi.com/article/10.3390/molecules30112433/s1, Figure S1. ^1^H NMR spectrum of IMesNHC–Cr(CO)_5_ (**1a**) in C_6_D_6_ solution; Figure S2. ^13^C NMR spectrum of IMesNHC–Cr(CO)_5_ (**1a**) in C_6_D_6_ solution; Figure S3. ^1^H NMR spectrum of IMesNHC–Mo(CO)_5_ (**2a**) in C_6_D_6_ solution; Figure S4. ^13^C NMR spectrum of IMesNHC–Mo(CO)_5_ (**2a**) in C_6_D_6_ solution; Figure S5. ^1^H NMR spectrum of IMesNHC–W(CO)_5_ (**3a**) in C_6_D_6_ solution; Figure S6. ^13^C NMR spectrum of IMesNHC–W(CO)_5_ (**3a**) in C_6_D_6_ solution; Figure S7. ^1^H NMR spectrum of IPrNHC–Cr(CO)_5_ (**1b**) in C_6_D_6_ solution; Figure S8. ^13^C NMR spectrum of IPrNHC–Cr(CO)_5_ (**1b**) in C_6_D_6_ solution; Figure S9. ^1^H NMR spectrum of IPrNHC–Mo(CO)_5_ (**2b**) in C_6_D_6_ solution; Figure S10. ^13^C NMR spectrum of IPrNHC–Mo(CO)_5_ (**2b**) in C_6_D_6_ solution; Figure S11. ^1^H NMR spectrum of IPrNHC–W(CO)_5_ (**3b**) in C_6_D_6_ solution; Figure S12. ^13^C NMR spectrum of IPrNHC–W(CO)_5_ (**3b**) in C_6_D_6_ solution; Table S1. Comparison of ^13^C NMR peaks in compounds NHC–M(CO)_5_ (NHC = IMesNHC, IPrNHC; M = Cr, Mo, W) in C_6_D_6_ with similar compounds from literature; Figure S13. Raman spectra of IMesNHC–Cr(CO)_5_ (**1a**), IMesNHC–Mo(CO)_5_ (**2a**), and IMesNHC–W(CO)_5_ (**3a**); Figure S14. Raman spectra of IPrNHC–Cr(CO)_5_ (**1b**), IPrNHC–Mo(CO)_5_ (**2b**), and IPrNHC–W(CO)_5_ (**3b**); Table S2. Calculated enthalpies (*H*) at the PBE/def2TZVP level of theory and calculated enthalpies of reactions Δ*H* in a.u. and kJ/mol; Table S3. Calculated Gibbs free energies (*G*) at the PBE/def2TZVP level of theory and calculated Gibbs free energies of reactions Δ*G* in a.u. and kJ/mol. Table S4. Selected crystal data for IMesNHC–Cr(CO)_5_ (**1a**) and IMesNHC–Mo(CO)_5_·MeCN (**2a**·MeCN); Table S5. Selected crystal data for IMesNHC–W(CO)_5_·MeCN (**3a**·MeCN) and IPrNHC–Mo(CO)_5_ (**2b**); Table S6. Selected bond lengths (Å) and bond angles (^o^) for IMesNHC–Cr(CO)_5_ (**1a**); Table S7. Selected bond lengths (Å) and bond angles (^o^) for IMesNHC–Mo(CO)_5_·MeCN (**2a**·MeCN); Table S8. Selected bond lengths (Å) and bond angles (^o^) for IMesNHC–W(CO)_5_·MeCN (**3a**·MeCN); Table S9. Selected bond lengths (Å) and bond angles (^o^) for IPrNHC–Mo(CO)_5_ (**2b**); Figure S15. Asymmetric unit of IMesNHC–Cr(CO)_5_·MeCN (**1a**·MeCN); Figure S16. Asymmetric unit of IMesNHC–Mo(CO)_5_·MeCN (**2a**·MeCN); Figure S17. Asymmetric unit of IMesNHC–W(CO)_5_·MeCN (**3a**·MeCN).
