# Supplementary material for: One-Pot Syntheses and Characterization of Group VI Carbonyl NHC Coordination Compounds
Source: Molecules. 2025 Jun 2;30(11):2433. doi: 10.3390/molecules30112433 (PMC12155831; doi:10.3390/molecules30112433)
Supplement: Supplementary file 1 [file molecules-30-02433-s001.zip › molecules-3659880-supplementary.pdf]

Supporting information

One-pot syntheses and characterization of Group VI carbonyl NHC coordination compounds

Zala Stopar, Evelin Gruden*, Melita Tramšek, Gašper Tavčar*

Department of Inorganic Chemistry and Technology, Jožef Stefan Institute, Jamova 39, 1000 Ljubljana, Slovenia

* Correspondence: gasper.tavcar@ijs.si (G.T.); evelin.gruden@ijs.si (E.G.)

Table of contents:

S1 NMR spectroscopy 2

S1.1 IMesNHC–$Cr(CO)_5$ (**1a**) 2

S1.2 IMesNHC–$Mo(CO)_5$ (**2a**) 3

S1.3 IMesNHC–$W(CO)_5$ (**3a**) 4

S1.4 IPrNHC–$Cr(CO)_5$ (**1b**) 5

S1.5 IPrNHC–$Mo(CO)_5$ (**2b**) 6

S1.6 IPrNHC–$W(CO)_5$ (**3b**) 7

S2 Raman spectroscopy 9

S3 Computational results 10

S4 Crystal Structure Data 12

S5 Crystal structures of selected compounds 17

S6 References 19

S1 NMR spectroscopy

S1.1 IMesNHC–Cr(CO)$_5$ (1a)

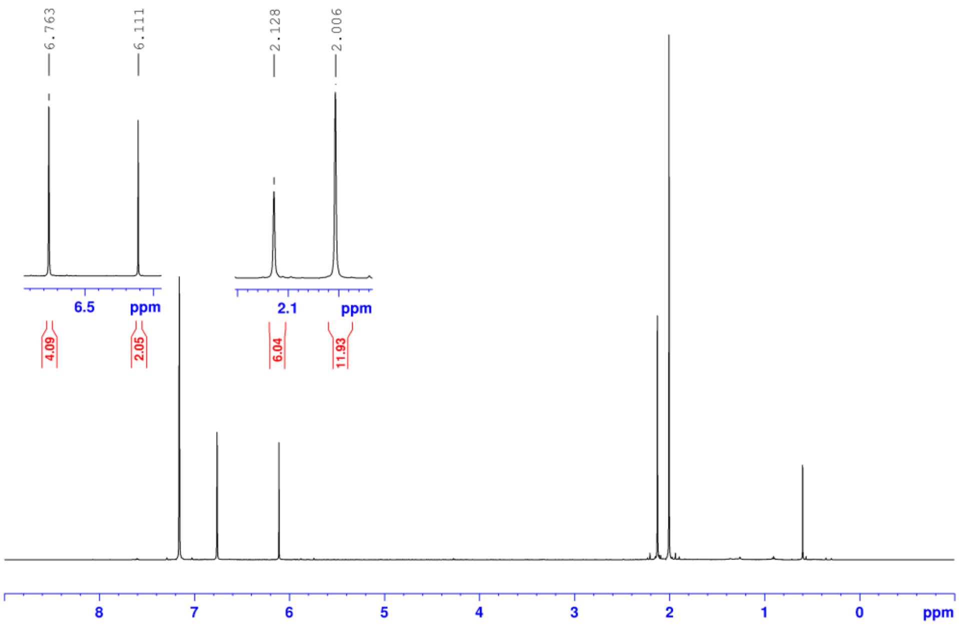

Figure S1. ^{1}H NMR spectrum of IMesNHC–Cr(CO)$_5$ (**1a**) in C_6D_6 solution.

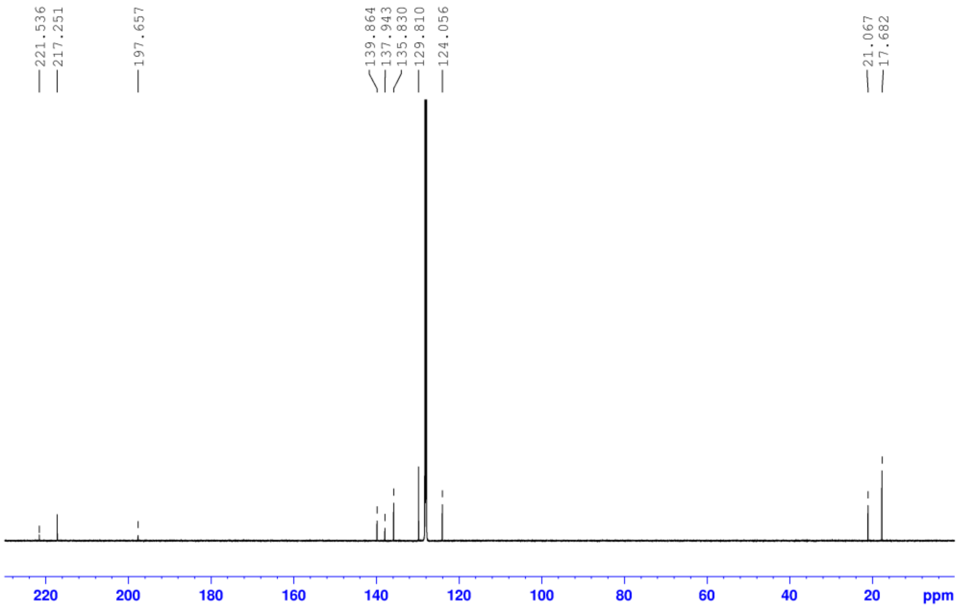

Figure S2. ^{13}C NMR spectrum of IMesNHC–Cr(CO)$_5$ (**1a**) in C_6D_6 solution.

S1.2 **IMesNHC–Mo(CO)$_5$ (2a)**

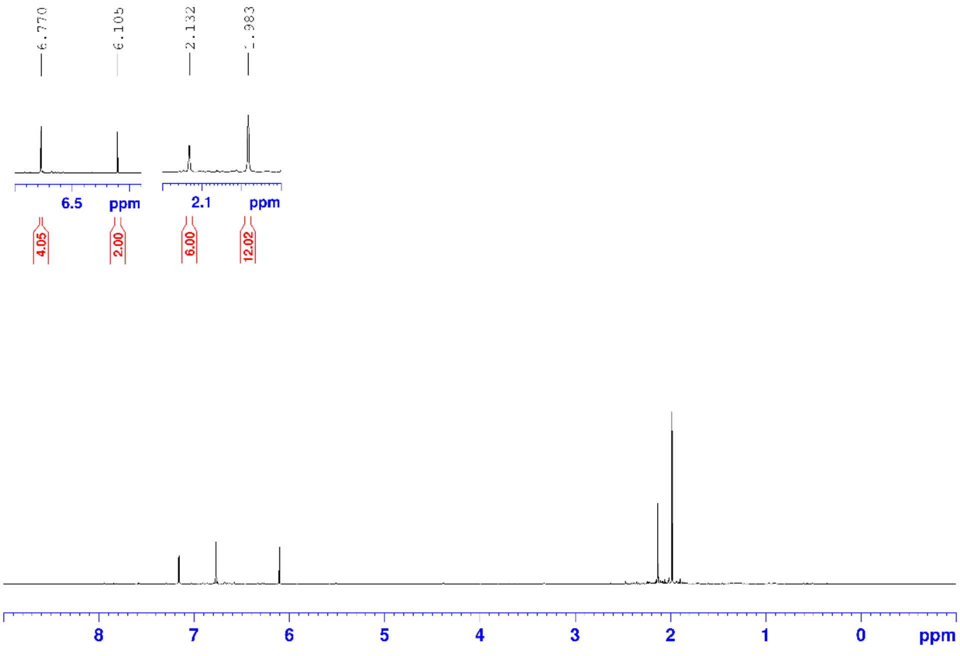

Figure S3. ^{1}H NMR spectrum of IMesNHC–Mo(CO)$_5$ (**2a**) in C_6D_6 solution.

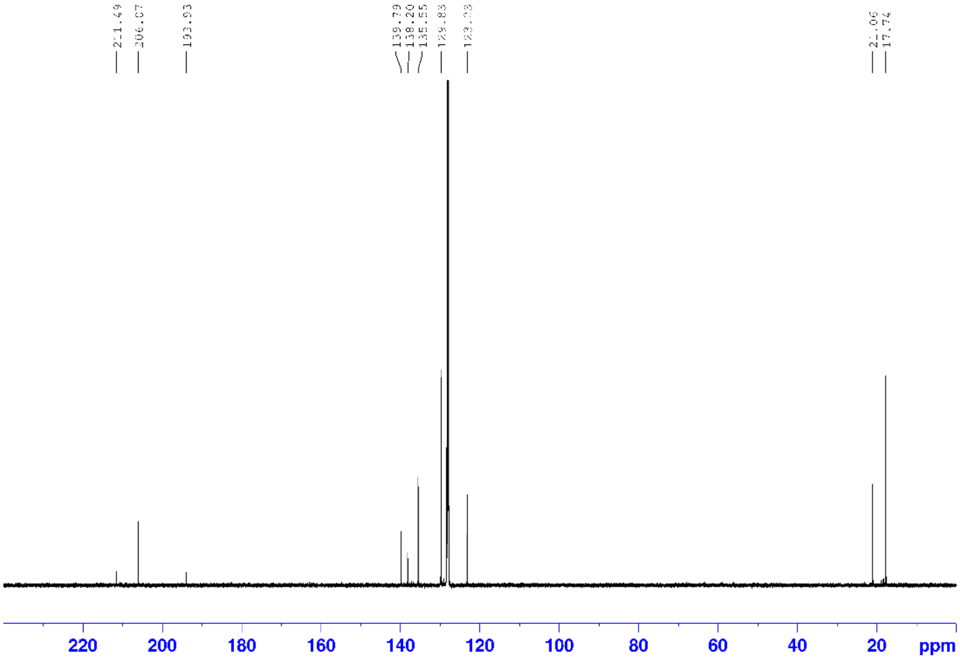

Figure S4. ^{13}C NMR spectrum of IMesNHC–Mo(CO)$_5$ (**2a**) in C_6D_6 solution.

S1.3 IMesNHC–W(CO)₅ (3a)

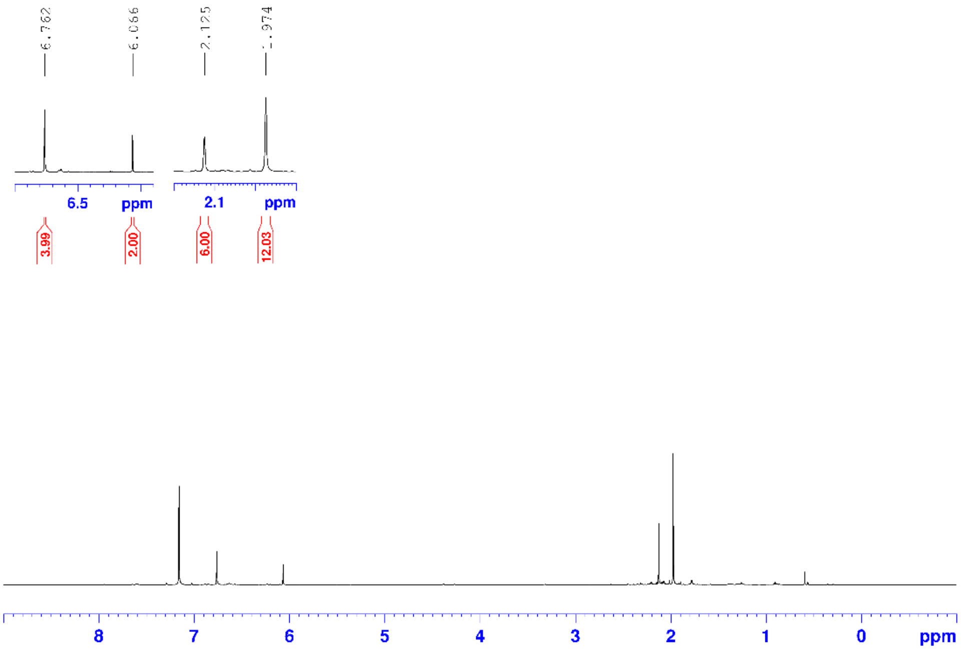

Figure S5. ^{1}H NMR spectrum of IMesNHC–W(CO)$_5$ (**3a**) in C_6D_6 solution.

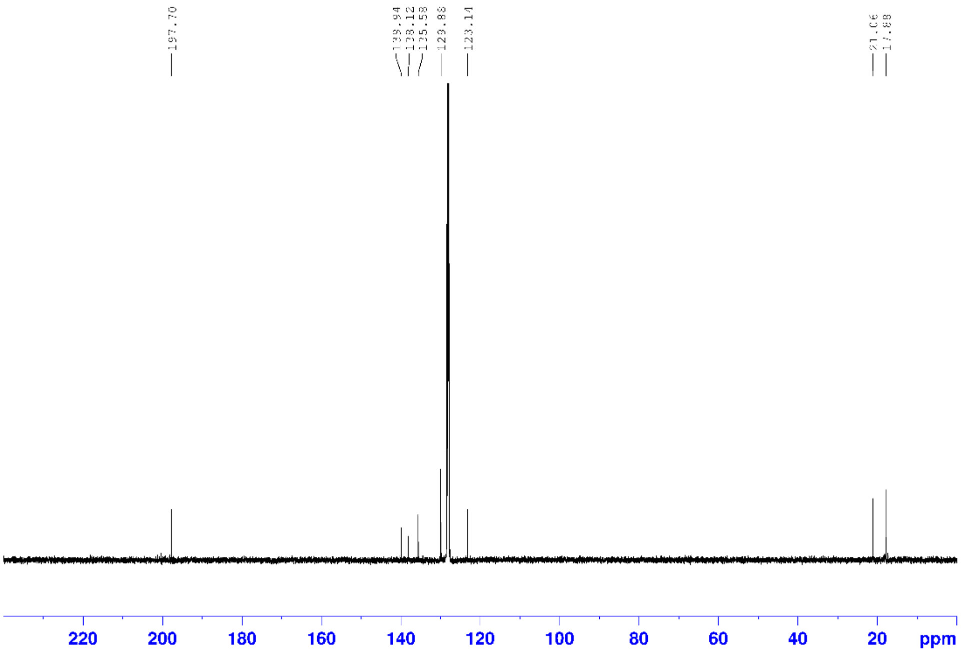

Figure S6. ^{13}C NMR spectrum of IMesNHC–W(CO)$_5$ (**3a**) in C_6D_6 solution.

S1.4 **IPrNHC–Cr(CO)$_5$ (1b)**

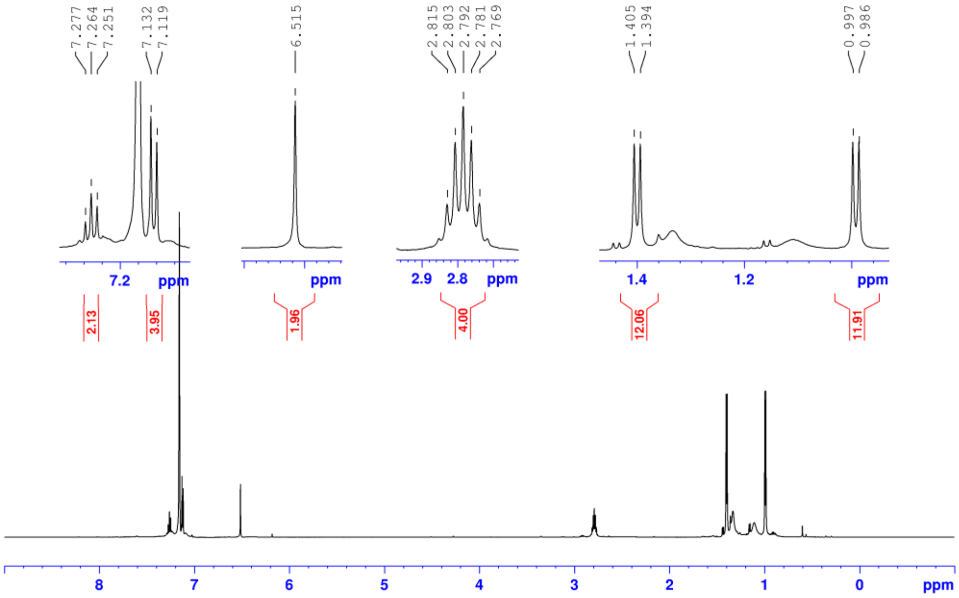

Figure S7. ^{1}H NMR spectrum of IPrNHC–Cr(CO)$_5$ (**1b**) in C_6D_6 solution.

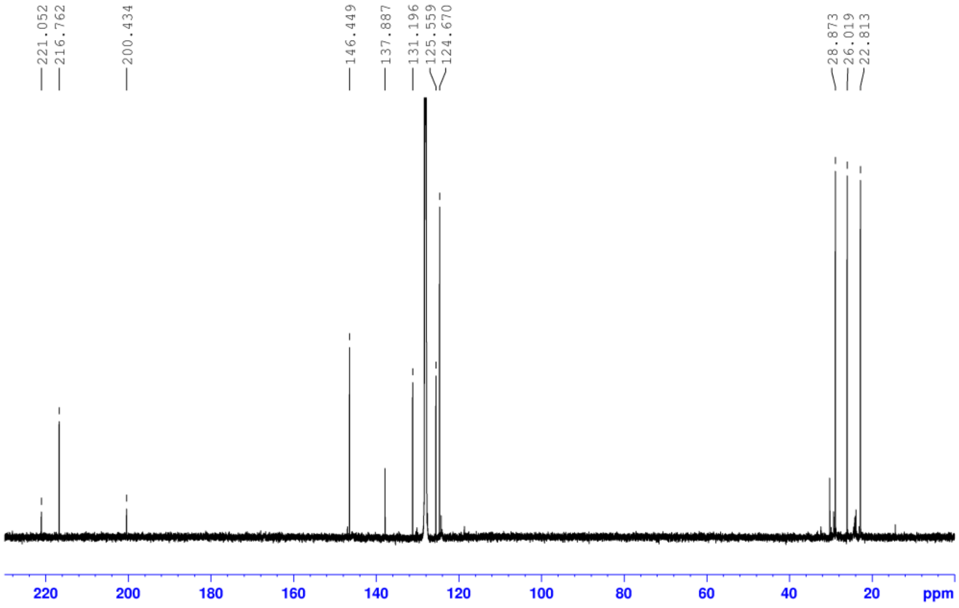

Figure S8. ^{13}C NMR spectrum of IPrNHC–Cr(CO)$_5$ (**1b**) in C_6D_6 solution.

S1.5 IPrNHC–Mo(CO)$_5$ (2b)

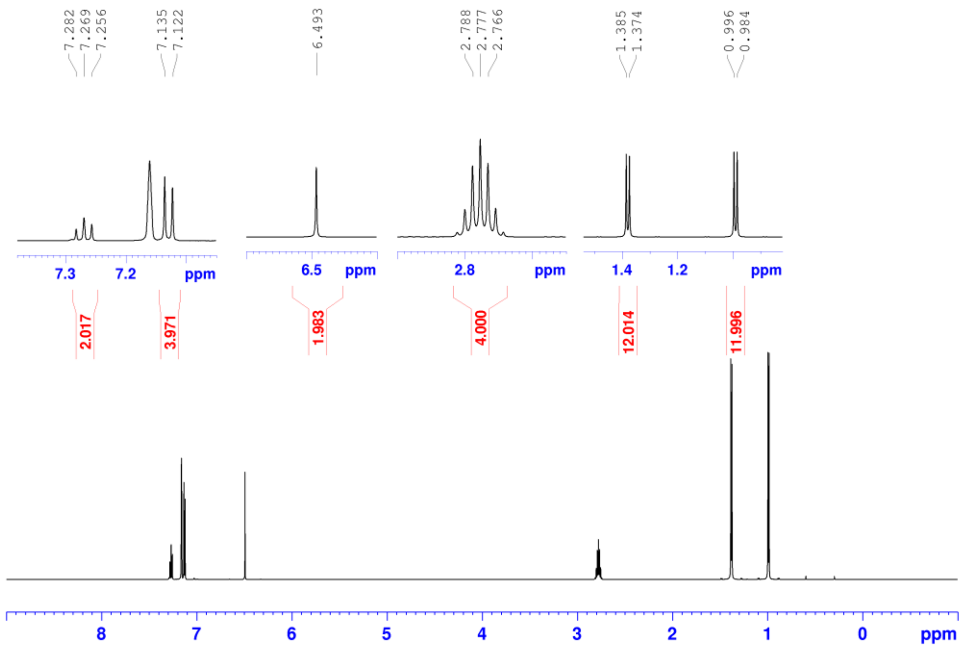

Figure S9. ^{1}H NMR spectrum of IPrNHC–Mo(CO)$_5$ (**2b**) in C_6D_6 solution.

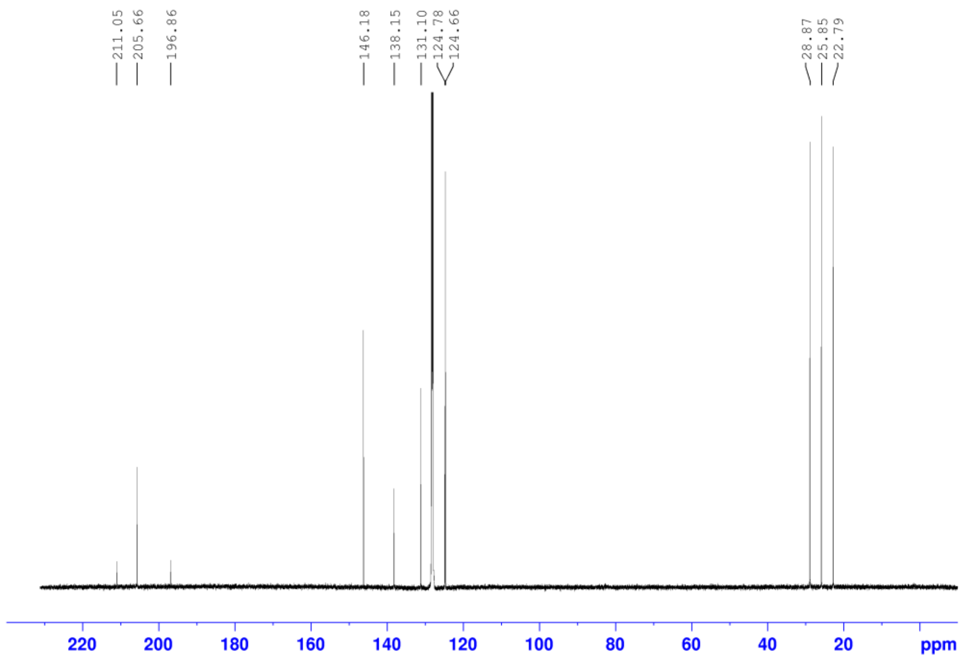

Figure S10. ^{13}C NMR spectrum of IPrNHC–Mo(CO)$_5$ (**2b**) in C_6D_6 solution.

S1.6 IPrNHC–W(CO)$_5$ (3b)

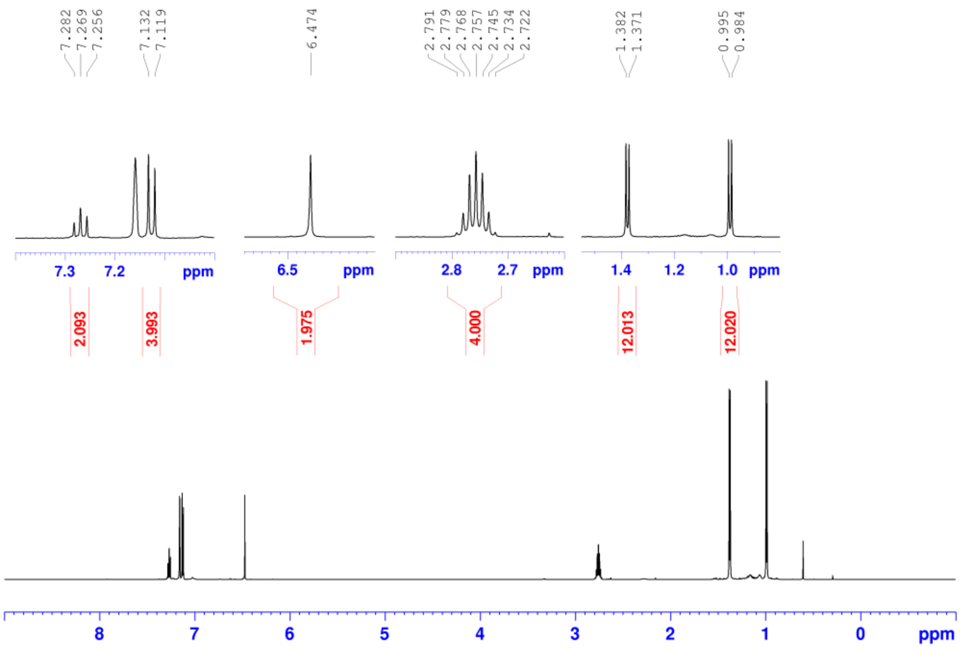

Figure S11. ^{1}H NMR spectrum of IPrNHC–W(CO)$_5$ (**3b**) in C_6D_6 solution.

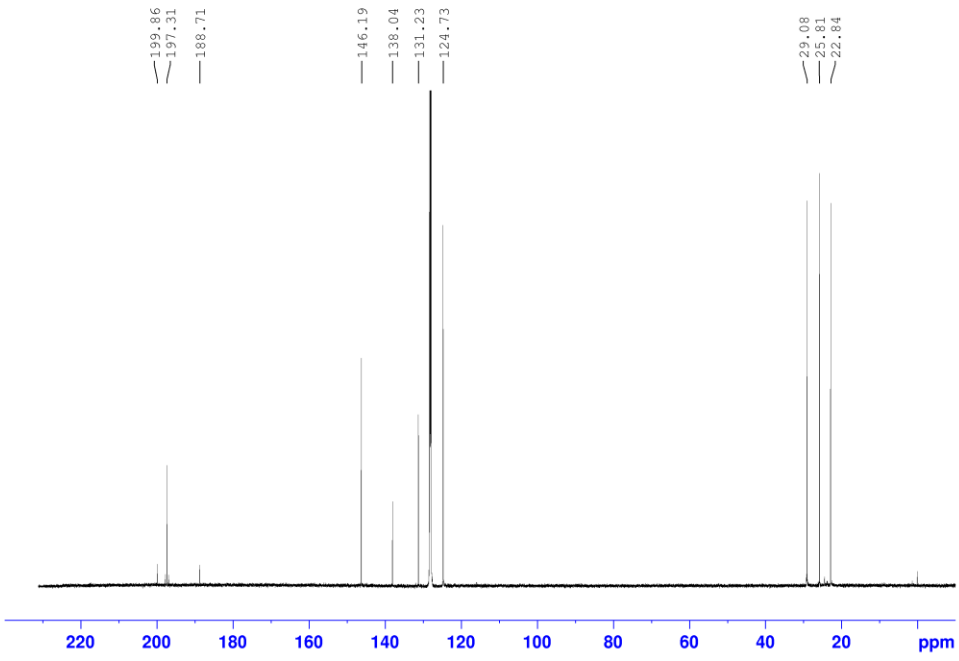

Figure S12. ^{13}C NMR spectrum of IPrNHC–W(CO)$_5$ (**3b**) in C_6D_6 solution.

Table S1. Comparison of ^{13}C NMR peaks in compounds NHC–M(CO)$_5$ (NHC = IMesNHC, IPrNHC; M = Cr, Mo, W) in C_6D_6 with similar compounds from literature.

**Compound**	δ (^{13}C) M–*C*O		δ (^{13}C) M–$\boldsymbol{C}_{(carbene)}$		
	Experimental	Literature	Experimental	Literature	Reference
IMesNHC–Cr(CO)$_5$ (**1a**)	221.5, 217.3	221.4, 216.6	197.7	197.3	[1]
IMesNHC–Mo(CO)$_5$ (**2a**)	221.5, 206.1	211.4, 205.3	193.9	193.6	[2]
IMesNHC–W(CO)$_5$ (**3a**)	197.7	200.4, 197.0	n.a.	185.6	[2]
IPrNHC–Cr(CO)$_5$ (**1b**)	221.0, 216.8	221.1, 216.1	200.4	199.9	[1]
IPrNHC–Mo(CO)$_5$ (**2b**)	211.0, 205.7	211.3, 205.1	196.9	196.3	[1]
IPrNHC–W(CO)$_5$ (**3b**)	199.9, 197.3	200.8, 197.2	188.7	187.8	[3]

S2 Raman spectroscopy

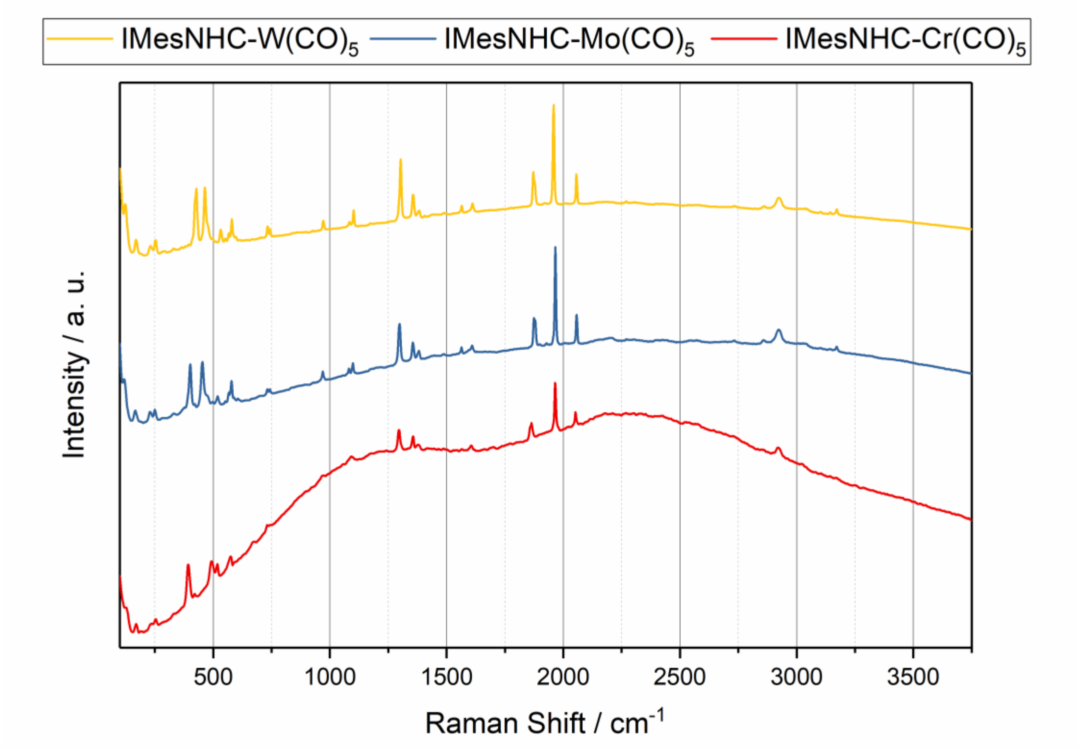

Figure S13. Raman spectra of IMesNHC–Cr(CO)$_5$ (**1a**), IMesNHC–Mo(CO)$_5$ (**2a**) and IMesNHC–W(CO)$_5$ (**3a**).

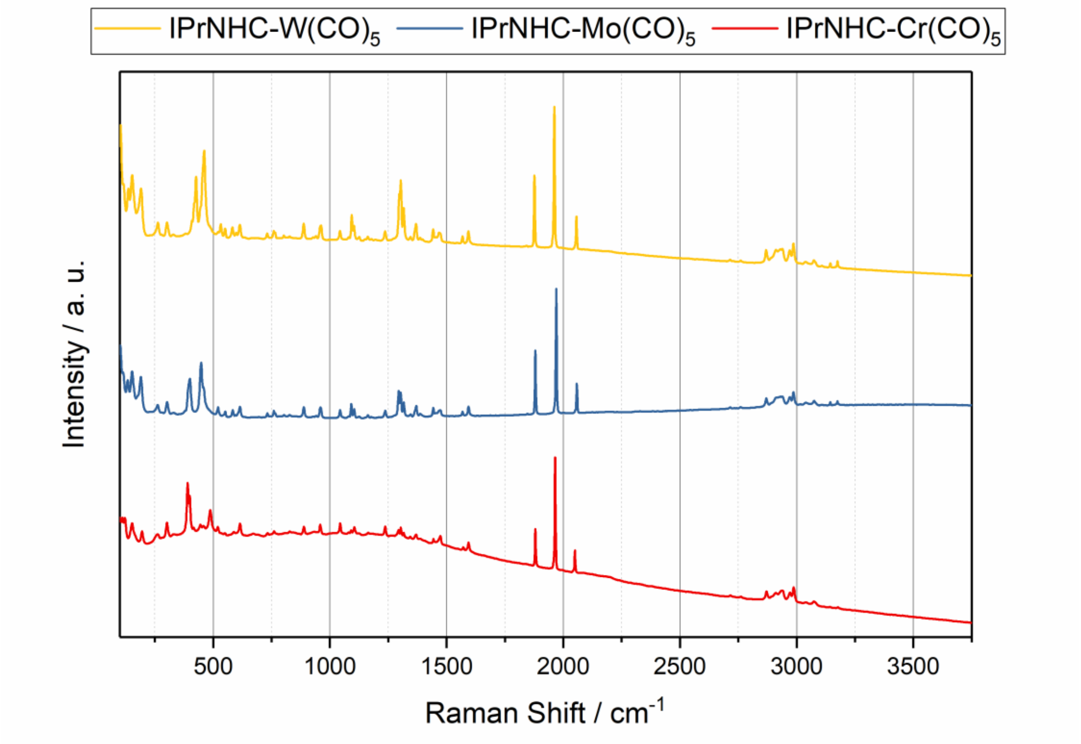

Figure S14. Raman spectra of IPrNHC–Cr(CO)$_5$ (**1b**), IPrNHC–Mo(CO)$_5$ (**2b**) and IPrNHC–W(CO)$_5$ (**3b**).

S3 Computational results

Table S2. Calculated enthalpies (*H*) at the PBE/def2TZVP level of theory and calculated enthalpies of reactions Δ*H* in a.u. and kJ/mol.

Compound	*H / a.u.*	Reaction	Δ*H / a.u.*	Δ*H* / kJ/mol
$Cr(CO)_6$	-1723.919653	/	/	/
$Mo(CO)_6$	-747.925067	/	/	/
$W(CO)_6$	-746.821280	/	/	/
IMesNHC	-922.938443	/	/	/
IPrNHC	-1158.395561	/	/	/
CO	-113.226545	/	/	/
IMesNHC–$Cr(CO)_5$	-2533.6552340	IMesNHC + $Cr(CO)_6$ → IMesNHC–$Cr(CO)_5$ + CO	-0.0236830	-62.18
IMesNHC–$Mo(CO)_5$	-1557.664482	IMesNHC + $Mo(CO)_6$ → IMesNHC–$Mo(CO)_5$ + CO	-0.0275170	-72.25
IMesNHC–$W(CO)_5$	-1556.561485	IMesNHC + $W(CO)_6$ → IMesNHC–$W(CO)_5$ + CO	-0.0283070	-74.32
IPrNHC–$Cr(CO)_5$	-2769.111627	IPrNHC + $Cr(CO)_6$ → IPrNHC–$Cr(CO)_5$ + CO	-0.0229580	-60.28
IPrNHC–$Mo(CO)_5$	-1793.122623	IPrNHC + $Mo(CO)_6$ → IPrNHC–$Mo(CO)_5$ + CO	-0.0285400	-74.93
IPrNHC–$W(CO)_5$	-1792.019729	IPrNHC + $W(CO)_6$ → IPrNHC–$W(CO)_5$ + CO	-0.0294330	-77.28

Table S3. Calculated Gibbs free energies (*G*) at the PBE/def2TZVP level of theory and calculated Gibbs free energies of reactions Δ*G* in a.u. and kJ/mol.

Compound	*G / a.u.*	Reaction	Δ*G / a.u.*	Δ*G* / kJ/mol
$Cr(CO)_6$	-1723.977526	/	/	/
$Mo(CO)_6$	-747.985472	/	/	/
$W(CO)_6$	-746.882044	/	/	/
IMesNHC	-923.021557	/	/	/
IPrNHC	-1158.490397	/	/	/
CO	-113.248985	/	/	/
IMesNHC–$Cr(CO)_5$	-2533.764962	IMesNHC + $Cr(CO)_6$ → IMesNHC–$Cr(CO)_5$ + CO	-0.0148360	-38.95
IMesNHC–$Mo(CO)_5$	-1557.778744	IMesNHC + $Mo(CO)_6$ → IMesNHC–$Mo(CO)_5$ + CO	-0.0175200	-46.00
IMesNHC–$W(CO)_5$	-1556.676019	IMesNHC + $W(CO)_6$ → IMesNHC–$W(CO)_5$ + CO	-0.0184660	-48.48
IPrNHC–$Cr(CO)_5$	-2769.233774	IPrNHC + $Cr(CO)_6$ → IPrNHC–$Cr(CO)_5$ + CO	-0.0148640	-39.03
IPrNHC–$Mo(CO)_5$	-1793.244404	IPrNHC + $Mo(CO)_6$ → IPrNHC–$Mo(CO)_5$ + CO	-0.0207000	-54.35
IPrNHC–$W(CO)_5$	-1792.141922	IPrNHC + $W(CO)_6$ → IPrNHC–$W(CO)_5$ + CO	-0.0214030	-56.19

S4 **Crystal Structure Data**

Table S4. Selected crystal data for IMesNHC–Cr(CO)$_5$ (**1a**) and IMesNHC–Mo(CO)$_5$·MeCN (**2a**·MeCN).

	IMesNHC–Cr(CO)$_5$ (**1a**)	IMesNHC–Mo(CO)$_5$·MeCN (**2a**·MeCN)
CCDC No.	2448749	2448748
Chemical formula	$C_{26}H_{24}CrN_2O_5$	$C_{28}H_{27}MoN_3O_5$
F_W (g/mol)	496.47	581.46
T (K)	150	150
λ (Å)	1.54184	1.54184
Crystal size (mm)	0.57 x 0.32 x 0.19	0.43 x 0.31 x 0.11
Crystal system	Monoclinic	Monoclinic
Space group	P21/n	P21/c
a (Å)	17.1956(3)	8.6053(2)
b (Å)	17.3891(2)	15.6382(3)
c (Å)	17.7965(3)	21.0346(3)
α (°)	90	90
β (°)	109.157(2)	101.407(2)
γ (°)	90	90
V (Å^3)	5026.8(2)	2774.74(9)
Z	8	4
q_{calc} (g/cm^3)	1.312	1.392
μ (mm^{-1})	4.061	4.206
F(000)	2064	1192
Θ range (°)	2.6–76.3	3.5–76.3
Index ranges	$-19 \le h \le 21$<br>$-21 \le k \le 21$<br>$-20 \le l \le 22$	$-10 \le h \le 10$<br>$-18 \le k \le 19$<br>$-26 \le l \le 24$
Reflections collected	34016	44450
Independent reflections	10413	5798
Reflections with (I > 2σ(I))	8216	5009
R_{int}	0.0316	0.0490
Data / restraints / parameters	10413 / 0 / 660	5798 / 0 / 360
S[a]	1.020	1.044
R_1[b], wR_2[c] (I > 2σ(I))	0.0407, 0.1052	0.0342, 0.0813
R_1[b], wR_2[c] (all data)	0.0582, 0.1204	0.0423, 0.0877
$\Delta\rho_{min}$, $\Delta\rho_{max}$ (e-Å^{-3})	–0.378, 0.368	–0.340, 0.623

[a] $S = [\Sigma(w(F_o^2-F_c^2)^2)/(N_o-N_p)]^{1/2}$.

[b] $R_1 = ||F_o|-|F_c||/\Sigma|F_o|$.

[c] $wR_2 = [\Sigma(w(F_o^2-F_c^2)^2)/\Sigma(w(F_o^2)^2)]^{1/2}$

Table S5. Selected crystal data for IMesNHC–W(CO)$_5$·MeCN (**3a**·MeCN) and IPrNHC–Mo(CO)$_5$ (**2b**).

	IMesNHC–W(CO)$_5$·MeCN (**3a**·MeCN)	IPrNHC–Mo(CO)$_5$ (**2b**)
CCDC No.	2448747	2448746
Chemical formula	$C_{28}H_{27}WN_3O_5$	$C_{32}H_{36}MoN_2O_5$
F_W (g/mol)	669.37	624.57
T (K)	150	150
λ (Å)	1.54184	0.71073
Crystal size (mm)	0.29 x 0.24 x 0.12	0.39 x 0.37 x 0.25
Crystal system	Monoclinic	Orthorhombic
Space group	P21/c	Cmcm
a (Å)	8.6040(2)	11.2283(5)
b (Å)	15.6386(2)	13.9658(7)
c (Å)	20.9235(3)	19.7145(9)
α (°)	90	90
β (°)	101.641(2)	90
γ (°)	90	90
V (Å^3)	2757.44(9)	3091.5(3)
Z	4	4
q_{calc} (g/cm^3)	1.612	1.342
μ (mm^{-1})	8.105	0.465
F(000)	1320	1296
Θ range (°)	2.8–76.3	3.6–28.8
Index ranges	$-10 \le h \le 10$<br>$-18 \le k \le 19$<br>$-26 \le l \le 26$	$-11 \le h \le 13$<br>$-15 \le k \le 16$<br>$-23 \le l \le 25$
Reflections collected	28855	7484
Independent reflections	5757	1875
Reflections with (I > 2σ(I))	4770	1766
R_{int}	0.0507	0.0284
Data / restraints / parameters	5757 / 0 / 360	1875 / 0 / 112
S[a]	1.050	1.102
R_1[b], wR_2[c] (I > 2σ(I))	0.0310, 0.0719	0.0228, 0.0539
R_1[b], wR_2[c] (all data)	0.0430, 0.0795	0.0251, 0.0555
$\Delta\rho_{min}$, $\Delta\rho_{max}$ (e Å^{-3})	–0.640, 0.1.227	–0.478, 0.284

[a] $S = [\Sigma(w(F_o^2-F_c^2)^2)/(N_o-N_p)]^{1/2}$.

[b] $R_1 = ||F_o|-|F_c||/\Sigma|F_o|$.

[c] $wR_2 = [\Sigma(w(F_o^2-F_c^2)^2)/\Sigma(w(F_o^2)^2)]^{1/2}$

Table S6. Selected bond lengths (Å) and bond angles (°) for IMesNHC–Cr(CO)$_5$ (**1a**).

Molecule 1		Molecule 2	
Bond lengths		Bond lengths	
Cr1–C12	2.132(2)	Cr2–C42	2.142(2)
Cr1–C1A	1.81(2)	C1A–O1A	1.19(2)
Cr1–C1B	1.89(3)	C1B–O1B	1.09(3)
Cr1–C2	1.898(3)	C2–O2	1.137(3)
Cr1–C3	1.907(2)	C3–O3	1.144(3)
Cr1–C4	1.907(3)	C4–O4	1.133(3)
Cr1–C5	1.913(3)	C5–O5	1.138(3)
Cr2–C6	1.847(2)	C6–O6	1.154(3)
Cr2–C7	1.896(2)	C7–O7	1.137(3)
Cr2–C8	1.900(2)	C8–O8	1.138(3)
Cr2–C9	1.917(2)	C9–O9	1.133(3)
Cr2–C10	1.915(2)	C10–O10	1.140(3)
Bond angles			
Cr1–C12–N11	128.6(2)	Cr2–C42–N41	128.4(2)
Cr1–C12–N13	128.8(1)	Cr2–C42–N43	128.8(2)
C1A–Cr1–C12	174.1(5)	Cr1–C1A–O1A	178.7(2)
C1B–Cr1–C12	173.6(7)	Cr1–C1B–O1B	178.(2)
C2–Cr1–C12	89.87(9)	Cr1–C2–O2	176.7(3)
C3–Cr1–C12	97.12(9)	Cr1–C3–O3	168.9(2)
C4–Cr1–C12	88.45(9)	Cr1–C4–O4	177.8(2)
C5–Cr1–C12	97.84(9)	Cr1–C5–O5	168.7(2)
C6–Cr2–C42	178.38(9)	Cr2–C6–O6	177.8(2)
C7–Cr2–C42	89.54(9)	Cr2–C7–O7	176.4(2)
C8–Cr2–C42	96.29(9)	Cr2–C8–O8	169.8(2)
C9–Cr2–C42	89.40(8)	Cr2–C9–O9	178.0(2)
C10–Cr2–C42	96.85(9)	Cr2–C10–O10	169.1(2)

Table S7. Selected bond lengths (Å) and bond angles (°) for IMesNHC–Mo$(CO)_5$·MeCN (**2a**·MeCN).

IMesNHC–Mo$(CO)_5$·MeCN (**2a**·MeCN)			
Bond lengths			
Mo1–C12	2.278(2)		
Mo1–C1	1.987(2)	C1–O1	1.149(3)
Mo1–C2	2.027(3)	C2–O2	1.134(4)
Mo1–C3	2.033(3)	C3–O3	1.137(4)
Mo1–C4	2.051(3)	C4–O4	1.132(4)
Mo1–C5	2.051(3)	C5–O5	1.131(4)
Bond angles			
Mo1–C12–N11	128.9(2)	Mo1–C12–N13	128.5(2)
C1–Mo1–C12	179.1(1)	Mo1–C1–O1	179.4(2)
C2–Mo1–C12	88.8(1)	Mo1–C2–O2	178.5(4)
C3–Mo1–C12	96.5(1)	Mo1–C3–O3	171.0(3)
C4–Mo1–C12	96.7(1)	Mo1–C4–O4	169.0(3)
C5–Mo1–C12	88.5(1)	Mo1–C5–O5	177.0(5)

Table S8. Selected bond lengths (Å) and bond angles (°) for IMesNHC–W$(CO)_5$·MeCN (**3a**·MeCN).

IMesNHC–W$(CO)_5$·MeCN (**3a**·MeCN)			
Bond lengths			
W1–C12	2.264(3)		
W1–C1	1.990(4)	C1–O1	1.151(5)
W1–C2	2.024(5)	C2–O2	1.151(6)
W1–C3	2.029(5)	C3–O3	1.137(6)
W1–C4	2.041(5)	C4–O4	1.135(6)
W1–C5	2.049(5)	C5–O5	1.134(7)
Bond angles			
W1–C12–N11	128.3(3)	W1–C12–N13	128.8(3)
C1–W1–C12	179.0(1)	W1–C1–O1	179.0(3)
C2–W1–C12	96.6(2)	W1–C2–O2	170.9(4)
C3–W1–C12	88.4(2)	W1–C3–O3	179.1(5)
C4–W1–C12	96.5(2)	W1–C4–O4	169.9(4)
C5–W1–C12	88.2(2)	W1–C5–O5	176.1(6)

Table S9. Selected bond lengths (Å) and bond angles (°) for IPrNHC–Mo(CO)$_5$ (**2b**).

IPrNHC–Mo(CO)$_5$ (**2b**)			
Bond lengths			
Mo1–C11	2.264(3)		
Mo1–C1	1.985(3)	C1–O1	1.1547(4)
Mo1–C2	2.050(2)	C2–O2	1.1394(3)
Mo1–C3	2.053(2)	C3–O3	1.1406(3)
Bond angles			
Mo1–C11–N12	128.9(1)		
C1–Mo1–C11	180.00	Mo1–C1–O1	180.00
C2–Mo1–C11	90.69(6)	Mo1–C2–O2	177.01(2)
C3–Mo1–C11	95.41(6)	Mo1–C3–O3	171.13(2)

S5 Crystal structures of selected compounds

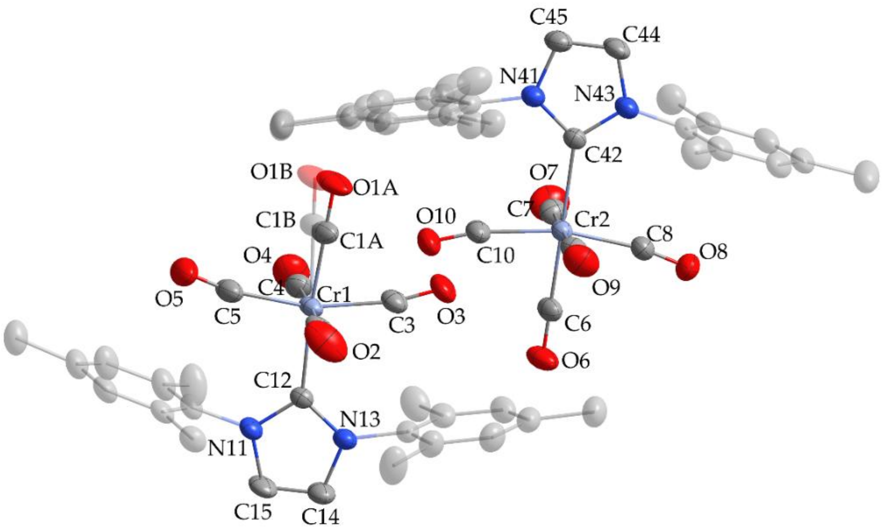

Figure S15. Asymmetric unit of IMesNHC–Cr(CO)$_5$·MeCN (**1a**·MeCN). The ellipsoids are drawn at 50% probability. The positions of the disordered atoms are shown in domain A and B. For clarity, all hydrogen atoms are omitted, while the domain B and the ''wingtips'' of the carbene are shaded.

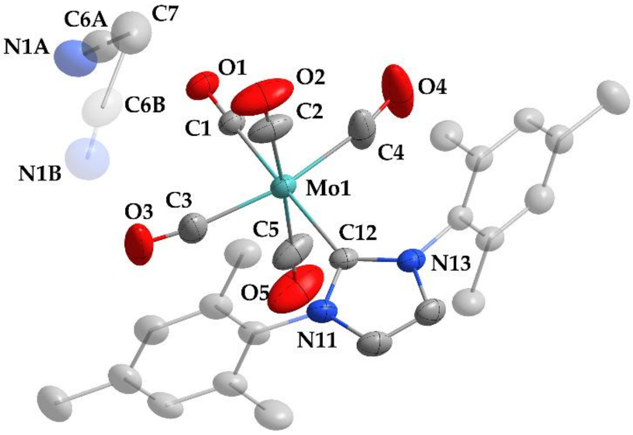

Figure S16. Asymmetric unit of IMesNHC–Mo(CO)$_5$·MeCN (**2a**·MeCN). The ellipsoids are drawn at 50% probability. For clarity, all hydrogen atoms are omitted and the ''wingtips'' of the carbene and the disordered MeCN are shaded. The positions of the disordered atoms are shown in domain A and B.

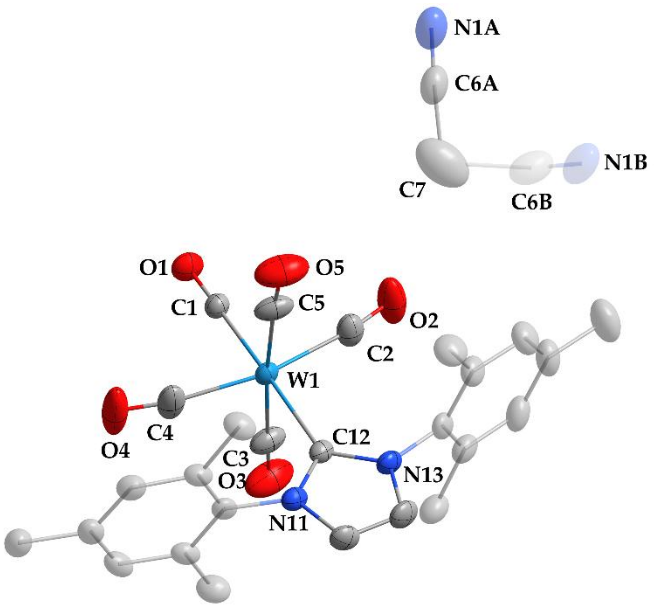

Figure S17. Asymmetric unit of IMesNHC–W(CO)$_5$·MeCN (**3a**·MeCN). The ellipsoids are drawn at 50% probability. For clarity, all hydrogen atoms are omitted and the ''wingtips'' of the carbene and the disordered MeCN are shaded. The positions of the disordered atoms are shown in domain A and B.

S6 References

1. Kim, S.; Choi, S.Y.; Lee, Y.T.; Park, K.H.; Sitzmann, H.; Chung, Y.K. Synthesis of Chromium *N*-Heterocyclic Carbene Complexes Using Chromium Fischer Carbenes as a Source of Chromium Carbonyls. *J. Organomet. Chem.* **2007**, *692*, 5390–5394, doi: 10.1016/j.jorganchem.2007.08.043.

2. Wang, Z.; Li, S.; Teo, W.J.; Poh, Y.T.; Zhao, J.; Hor, T.S.A. Molybdenum (0) and Tungsten (0) Carbonyl *N*-Heterocyclic Carbene Complexes as Catalyst for Olefin Epoxidation. *J. Organomet. Chem.* **2015**, *775*, 188–194, doi: 10.1016/j.jorganchem.2014.07.009.

3. Ghadwal, R.S.; Rottschäfer, D.; Andrada, D.M.; Frenking, G.; Schürmann, C.J.; Stammler, H.-G. Normal-to-Abnormal Rearrangement of an N-Heterocyclic Carbene with a Silylene Transition Metal Complex. *Dalton Trans.* **2017**, *46*, 7791–7799, doi: 10.1039/C7DT01199G.
